# Immunomodulation for treatment of drug and device refractory gastroparesis

**DOI:** 10.1016/j.rinim.2016.02.001

**Published:** 2016-03-03

**Authors:** Kaartik Soota, Archana Kedar, Yana Nikitina, Evelyn Arendale, Vetta Vedanarayanan, Thomas L. Abell

**Affiliations:** a Department of Internal Medicine, Unity Health System, Rochester, NY, United States; b Digestive Diseases, University of Mississippi Medical Center, Jackson, MS, United States; c Pediatric Neurology, University of Mississippi Medical Center, Jackson, MS, United States; d Digestive Diseases, University of Louisville Medical Center, Louisville, KY, United States

**Keywords:** Autonomic diseases, Autoimmune diseases, Gastrointestinal, Neuromuscular disease

## Abstract

**Objective:**

Patients with generalized autoimmune dysautonomia may also present with gastroparesis. Immune dysfunction in such patients can be evaluated using antibodies to glutamic acid decarboxylase (GAD) and full thickness biopsy of stomach. In this study, we utilize immunotherapy for treatment of drug and Gastric Electrical Stimulation (GES) resistant gastroparetic patients with evidence of neuroinflammation on full thickness gastric biopsy and had positive GAD65 autoantibodies.

**Material and methods:**

We conducted a retrospective chart review of 11 female patients with drug and device resistant gastroparesis. Patients were treated for a total of 8–12 weeks with either intravenous immunoglobulin (IVIg), or combined mycophenolate mofetil (MM) and methylprednisolone, or only MM. Patients were excluded if they had previous side effects from steroid therapy, low scores on dual-energy X-ray absorptiometry (DEXA) scan results, immune-compromised conditions with infections like tuberculosis and zoster. Symptoms of nausea, vomiting, abdominal pain, early satiety/anorexia, bloating and total symptom score (TSS) as reported by the patients were recorded before and after the treatment at a follow up visit 2 to 16 weeks after initiation of therapy.

**Results:**

Maximum symptom improvement was seen in patients treated with IVIg (67%). 6 patients (55%) had improvement in vomiting, whereas 5 patients (45%) had improvements in nausea, abdominal pain and bloating.

**Conclusions:**

Immunomodulatory therapy shows positive outcomes in improving vomiting symptom in some gastroparetic patients who have coexisting positive autoimmune profiles. This preliminary data suggests the need for further investigations in immunotherapy targeted to patients with gastroparetic symptoms refractory to approved drug and device therapies.

## 1 Introduction

Gastrointestinal motility disorders can be found in the setting of generalized autoimmune dysautonomia and may present as gastroparesis [1]. Several antibodies have been associated with autoimmune disorders presenting with symptoms of gastroparesis [2]. Antibodies to Glutamic Acid Decarboxylase (GAD) have been described in Type 1 diabetes mellitus, and anti-GAD65 is the most studied isoform [3], [4]. Antibodies to GAD have also been extensively studied in many other autoimmune disorders like Stiff-Person Syndrome (SPS) and Dermatomyositis [5], [6]. High titers of GAD antibodies signify autoimmune dysfunction and the use of immunomodulating drugs may be indicated for symptomatic patients with these markers. Studies have shown the benefits of immunomodulatory therapy in such conditions; however, it has been systematically applied only in SPS [5].

Immune dysfunction in gastroparesis is also evident on full thickness biopsy of the stomach. Patients with gastroparesis show increased CD45 immunofluorescence in the myenteric plexus and increased CD68 infiltration in the muscle layers, significant of increased immune cells and macrophages expression, respectively, in the stomach [7]. Along with evidence of immune dysfunction, gastroparetic patients also have decreased number of interstitial cells of Cajal and decreased nerve fibers [7].

Immunotherapy has been utilized for patients with autonomic neurological disorders. Using the same concept, immunotherapy trial has been utilized with good results for evaluation of patients with presumed autoimmune gastrointestinal dysmotility [8]. In this study, we utilize immunotherapy for treatment of drug and GES resistant patients with symptoms of gastroparesis who were positive for gastric enteral neuroinflammation on full thickness gastric biopsy and had positive GAD65 autoantibodies.

## 2 Methods

### 2.1 Study design

This is a retrospective case series of patients identified through medical chart review. The clinical protocol was reviewed and approved by the University of Louisville Institutional Review Board.

### 2.2 Study subjects

We included gastroparetic patients who had sub-optimal response to medical therapy and/or gastric enteral stimulation therapy. All patients had evidence of neuroinflammation as defined by positive GAD65 antibodies and presence of inflammatory markers on full thickness gastric biopsy. Western blotting (ANABLOT) was used to identify human auto-antibodies present in serum with specificity for a number of antibodies including Scl 70, Scl 105, SSB 43, Sm 16, Sm 18, and Ku 66. A scaled GI banding score or GIBS was used to standardize the bands present. All patients also had evaluation of paraneoplastic antibody panel which included antineuronal nuclear autoantibody type 1, 2, and 3 (ANNA-1, ANNA-2, ANNA-3), anti-glial nuclear antibody (AGNA), Purkinje Cell Cytoplasmic Antibody Type-1 (PCA-1), Purkinje Cell Cytoplasmic Antibody Type-2(PCA-2), Purkinje Cell Cytoplasmic Antibody Type Tr (PCA-Tr), Amphiphysin Antibody, CRMP-5 antibody, Striated Muscle antibody, P/Q-Type Calcium Channel Antibody, N-Type Calcium Channel Antibody, ACh Receptor (Muscle) Binding Antibody, AChR Ganglionic Neuronal Antibody, Neuronal (V-G) K+ Channel Antibody. Patient exclusion criteria included: previous side effects from steroid therapy, low scores on dual-energy X-ray absorptiometry (DEXA) bone scan results, immune-compromised conditions with infections such as tuberculosis and zoster.

## 3 Methods

All patients in the study received therapy with immunosuppressive therapy with either intravenous immunoglobulin, or mycophenolate mofetil (MMF), or combined daily mycophenolate mofetil according to the protocol at our center. Intravenous immunoglobulin or IVIg (this medication is available by a number of trade names: Carimune NF-BDI Pharma, Columbia, SC; Flebogamma 5% DIF-Grifols, Los Angeles, CA; Gamunex 10%-Talecris, Durham, NC; Gammagard S/D-Baxter, Deerfield, IL; Octagam 5%-Octapharma, Toronto, ON) was given weekly for 8–12 weeks. Mycophenolate Mofetil or MMF (CellCept, Genentech, South San Francisco, California) was given daily for 12 weeks. Combined MMF and intravenous or oral methylprednisolone (Medrol, Pfizer, New York, New York) were given daily for 8–12 weeks. Symptoms of nausea, vomiting, abdominal pain, early satiety/anorexia, and bloating as reported by the patients on a 5 point scale of 0–4 with 4 being the worst, were recorded before and after the treatment. Total symptom score (TSS) was calculated by addition of all the symptom scores. All patients were continued on their medications for symptomatic management of gastroparesis and had their GES turned ON. Patients were followed up at clinical visit or by phone call from 2 to 16 weeks after the initiation of therapy to monitor the response with final determination of therapeutic response performed after at least 12 weeks of therapy.

## 4 Results

Our clinical series included 11 female gastroparesis patients (10 Caucasians, 1 African-American) with mean of age 45 years. Ten patients had a history of idiopathic gastroparesis and 1 patient had diabetic gastroparesis. Three patients were treated with IVIg, four with combined methylprednisolone and mycophenolate mofetil, and four with mycophenolate mofetil only (Table 1). ANABLOT performed on 8 patients showed 7 patients with more than 3 bands each giving them GIBS≥3 (normal<3). Paraneoplastic antibodies were found in 2 patients. Symptom scores were recorded in all the 11 patients (Table 2). Among the patients who received only IVIg, 1 out of 3 had improved vomiting, 2 of 3 had improved nausea, abdominal pain, bloating, and TSS. For the patients who received combined treatment with mycophenolate mofetil and methylprednisolone, 2 out of 4 patients had improvement in vomiting, 1 of 2 had improvement in nausea, bloating and TSS. In the group of patients who received only mycophenolate mofetil, vomiting improved in 3 of 4 patients; nausea, abdominal pain and bloating improved in 2 of 4 patients whereas TSS also improved in 3 of 4 patients. All patients combined together, 6/11 (55%) had improvement in vomiting whereas 5/11 (45%) had improvements in nausea, abdominal pain and bloating. TSS improved in 6 of 11 (55%) patients. Maximum improvement was seen in the patients receiving IVIg as 2/3 patients (67%) had improved TSS with largest improvement seen with symptoms of nausea, abdominal pain and bloating.

## 5 Discussion

This is a pilot study evaluating the role of immunotherapy in patients with drug and GES device refractory gastroparesis with evidence of autoimmune dysfunction. In our study we have shown that treatment with immunotherapy in such patients resulted in symptom improvement in more than 50% of patients as measured by TSS. We also showed that maximum benefit is obtained by utilizing IVIg as the immunomodulatory agent.

Gastroparesis is a complex clinical entity, many aspects of which still remain unknown requiring further investigations [1]. Most patients have idiopathic (36%), diabetic (29%) or post-surgical (13%) gastroparesis, however, other causes like autoimmune, paraneoplastic and neurological, are also present. Medical therapy is limited to prokinetic and anti-emetic drugs to relieve the gastroparetic symptoms of nausea, vomiting, abdominal pain, early satiety or bloating. Gastric electrical stimulator (GES) can be used in drug refractory gastroparesis and has been shown to be an effective treatment for gastroparesis, and intractable nausea and vomiting but its effects are limited and many times inadequate [1], [9]

In our study, vomiting was the most improved symptom (in 55% of patients) with nausea, abdominal pain and bloating closely behind (in 45% patients). Patients receiving IVIg reported the maximum benefit (in 66% patients). Flanagan et al. recently utilized immune therapy to diagnose autoimmune gastrointestinal dysmotility, although the same study also mentioned about the improvement of symptoms in such patients [8]. The same study utilized IVIg on all their patients (*n*=23) of which 17 had favorable response (73%). Our study had a response rate of 66% which is similar to the other study. However, there are few important differences between the two studies. Along with serological testing, we also utilized full thickness gastric biopsy and Western blot of anti-nuclear antibody (ANAblot) profile to define the presence of immune dysfunction in the patient necessitating immune therapy. On the other hand, Flanagan et al. conducted autonomic testing on all of the patients which was not performed in all of the patients in our study. Both the reports performed similar neural antibody testing.

It should be noted that despite the diagnosis of immune dysfunction leading to idiopathic gastroparesis in all the patients, only two-thirds of the patients responded well to the treatment. There is no clear explanation for this but it definitely needs to be evaluated more in the future so that we can utilize this therapy in selected patients only to prevent unnecessary exposure to medications. Also, our study is a retrospective case series with limited number of patients and does not have a placebo control group to compare the results; however, the results are encouraging and suggest that, if appropriately diagnosed, gastroparesis in the setting of immune dysfunction can effectively be managed with immune therapy.

In conclusion, to our knowledge, this is the first report of the use of immunotherapy for treatment of drug and GES resistant gastroparesis, when GES was utilized. This pilot study suggests that this technique should be considered as a part of the armamentarium against gastroparesis. However, to further validate the potential clinical benefit, we recommend prospective evaluation of this therapy in randomized controlled and blinded trials.

## Conflicts of interest

None.

## Source of funding

None.

## Figures and Tables

**Table 1:** Baseline antibody profiles of gastroparesis patients selected for immunotherapy treatment.

Patient no.	Immunotherapy treatment	ANABLOT (no. of bands)	Paraneoplastic Antibodies	GAD65
1	Mycophenolate mofetil	5	Negative	Positive
2	Mycophenolate mofetil and Methylprednisolone	5	Negative	Positive
3	Mycophenolate mofetil and Methylprednisolone	4	Negative	Positive
4	Mycophenolate mofetil	7	Negative	Positive
5	Mycophenolate mofetil	2	0.03	Positive
6	Mycophenolate mofetil and Methylprednisolone	6	Negative	Positive
7	Mycophenolate mofetil and Methylprednisolone	JO-1–0.6	Negative	Positive
8	Mycophenolate mofetil	7	Negative	Positive
9	Immunoglobulin	JO-1–0.3	Negative	Positive
10	Immunoglobulin	JO-1–1	0.03	Positive
11	Immunoglobulin	4	Negative	Positive

GAD65: antibodies to glutamic acid decarboxylase, isoform 65.

JO-1: antibodies to cytoplasmic protein, histidyl tRNA.

ANABLOT: western blot for human auto-antibodies including Scl 70, Scl 105, SSB 43, Sm 16, Sm 18, and Ku 66.

Paraneoplastic antibodies: ANNA-1, ANNA-2, ANNA-3, AGNA, PCA-1, PCA-2, PCA-Tr, Amphiphysin Antibody, CRMP-5 antibody, Striated Muscle antibody, P/Q-Type Calcium Channel Antibody, N-Type Calcium Channel Antibody, ACh Receptor (Muscle) Binding Antibody, AChR Ganglionic Neuronal Antibody, Neuronal (V-G) K+ Channel Antibody.

**Table 2:** Patient reported change in symptoms after finishing immunotherapy (n represents the number of patients in each group).

Symptoms	Immunoglobulin therapy (*n*=3)			MMF Therapy (*n*=4)			MMF+Methylprednisolone therapy (*n*=4)		
	Improvement	No change	Worsening	Improvement	No change	Worsening	Improvement	No Change	Worsening
Nausea	2	1	0	2	1	1	1	1	2
Vomiting	1	1	1	3	1	0	2	0	2
Abdominal pain	2	1	0	2	1	1	1	2	1
Anorexia/Early satiety	0	2	1	1	1	1	1	3	0
Bloating	2	1	0	2	1	1	1	1	2
TSS	2	1	0	3	1	0	1	1	2

TSS: Total symptom score.
